# Robotic Hand–Eye Calibration Method Using Arbitrary Targets Based on Refined Two-Step Registration

**DOI:** 10.3390/s25102976

**Published:** 2025-05-08

**Authors:** Zining Song, Chenglong Sun, Yunquan Sun, Lizhe Qi

**Affiliations:** Intelligent Robotics Research Institute, Fudan Academy for Engineering and Technology, Fudan University, Shanghai 200433, China; 22210860110@m.fudan.edu.cn (Z.S.); clsun23@m.fudan.edu.cn (C.S.)

**Keywords:** hand–eye calibration, 3-dimention measurement, RANSAC, robotic

## Abstract

To optimize the structure and workflow of the 3D measurement robot system, reduce the dependence on specific calibration targets or high-precision calibration objects, and improve the versatility of the system’s self-calibration, this paper proposes a robot hand–eye calibration algorithm based on irregular targets. By collecting the 3D structural information of an object in space at different positions, a random sampling consistency evaluation based on the fast point feature histogram (FPFH) is adopted, and the iterative closest point (ICP) registration algorithm with the introduction of a probability model and covariance optimization is combined to iteratively solve the spatial relationship between point clouds, and the hand–eye calibration equation group is constructed through spatial relationship analysis to solve the camera’s hand–eye matrix. In the experiment, we use arbitrary objects as targets to execute the hand–eye calibration algorithm and verify the effectiveness of the method.

## 1. Introduction

Robot-based 3D measurement systems have been widely applied in quality inspection [1,2,3,4,5] and reverse engineering [6,7]. Among these systems, hand–eye calibration is an essential initialization step, where its accuracy significantly impacts the final measurement precision [8].

Current 3D camera calibration methods primarily rely on specific targets, including 2D patterns with distinctive features and specially designed 3D targets [9]. For 2D targets, notable methods include Tsai’s calibration approach based on radial alignment constraints using planar targets with known 3D structures [10], Zhang’s flexible camera calibration technique using planar patterns [11], as well as target designs based on Zhang’s method such as ARToolkit [12], ARTag [13], and AprilTag [14] calibration boards. Regarding 3D targets, designs include standard spherical targets [15] and those with specific geometric features [16,17,18,19].

Although target-based calibration methods demonstrate excellent accuracy and stability, their performance depends heavily on the manufacturing precision of the targets themselves, requiring specific designs and preparations while being sensitive to environmental variations. Consequently, researchers have recently explored target-less calibration approaches. Reference [20] proposed a method using arbitrary free-form surfaces, estimating hand–eye parameters through feature matching between measurement data and design models. Reference [21] introduced a scene-feature-based approach that computes camera intrinsics and hand–eye relationships by extracting ORB features from consecutive scene images. However, this method depends on the features contained in depth images and imposes strict requirements on capture range and motion sequence continuity.

To enhance the robustness of robot hand–eye calibration and reduce dependence on high-precision targets and controlled environments, this paper presents a novel calibration method using unfixed 3D objects. Our approach employs arbitrary objects of appropriate size as 3D targets, utilizing a two-step improved ICP algorithm with RANSAC-based feature recognition. By performing local registration of the target from different viewpoints, we establish relative relationships between target positions in various camera frames. Through the comprehensive modeling of the hand–eye system and the introduction of a virtual camera coordinate origin, we transform target relationships into inter-camera transformations, constructing the parameters required by traditional calibration methods to solve for high-accuracy hand–eye matrices.

This research implements robot hand–eye calibration by establishing coordinate transformations of non-fixed 3D targets through registration based on RANSAC feature recognition and improved ICP algorithms. Section 2.3 introduces the calibration methodology and core algorithms. Section 2.4 introduces the multi-step registration algorithm. Section 3 presents experiments using arbitrary targets, accuracy validation tests, and detailed error analysis. Section 4 summarizes the paper, discusses the limitations of the current study, and outlines potential future research directions.

## 2. Methods

### 2.1. Hand–Eye Robot System

**Figure 1:**
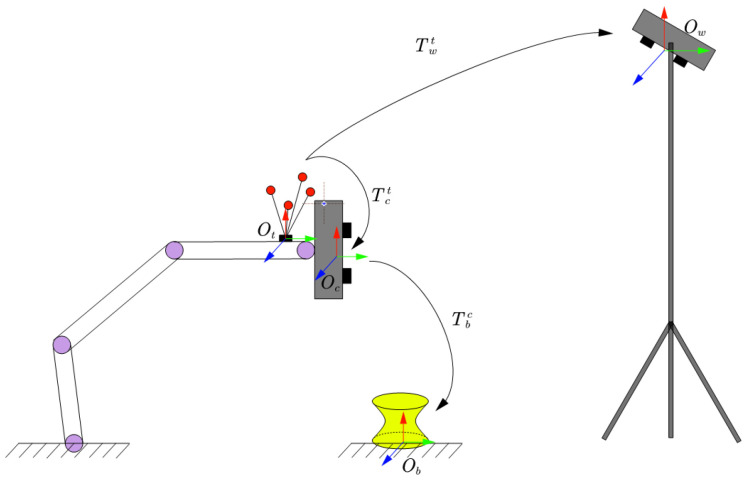
Measurement system.

Based on the position of the camera used to acquire calibration information—whether it is mounted on the robot’s end-effector or external to the robot—robot hand–eye systems can be classified into two categories as follows: Eye-In-Hand and Eye-to-Hand. The system designed in this paper includes both a structured light camera mounted on the robot’s end-effector to capture target information and a binocular tracking camera to obtain the pose of the robot’s end-effector. Therefore, this system does not fall into either of these two typical categories. As shown in Figure 1, the hand–eye system used in this paper consists of the following components:

- Fixed binocular tracking camera (W): Used to track the robot’s end-effector;
- Tracking target (T): Mounted on the robot’s end-effector, tracked by the binocular camera;
- Structured light 3D scanner (C): Mounted on the robot’s end-effector, used to capture 3D information of the target;
- Arbitrary 3D target (B): Used as the calibration object.

This system integrates both internal and external sensing capabilities, enabling robust and flexible calibration without relying on a fixed target or a single camera configuration.

In this system, the coordinate systems are defined as follows: $\left\{O_{b}\right\}$: The fixed arbitrary 3D target coordinate system. $\left\{O_{t}\right\}$: The tracking target coordinate system attached to the robot’s end-effector. $\left\{O_{c}\right\}$: The structured light 3D camera coordinate system. $\left\{O_{w}\right\}$: The fixed world coordinate system of the tracking camera.

The transformation matrices are defined as follows: $T_{t}^{w}$: The homogeneous transformation matrix from the tracking target coordinate system $\left\{O_{t}\right\}$ to the world coordinate system $\left\{O_{w}\right\}$, obtained by the tracking camera. $T_{c}^{t}$: The homogeneous transformation matrix from the tracking target coordinate system $\left\{O_{t}\right\}$ to the structured light camera coordinate system $\left\{O_{c}\right\}$ that needs to be calibrated. $T_{b}^{c}$: The homogeneous transformation matrix from the arbitrary 3D target coordinate system $\left\{O_{b}\right\}$ to the structured light camera coordinate system $\left\{O_{c}\right\}$.

When the measurement system is operational, a point $p_{c}^{i}$ measured in the structured light camera coordinate system is transformed into a point $p_{w}^{i}$ in the world coordinate system using the following transformation:

(1) $$p_{w}^{i}=T_{t_{i}}^{w}T_{c}^{t}p_{c}^{i}$$

Here, $T_{t_{i}}^{w}$ represents the pose of the rigid body composed of the structured light camera and its attached tracking target in the world coordinate system at the time of measurement. By unifying the measurement results from the structured light camera at different positions and orientations, the final complete measurement result is obtained.

This approach ensures that the measurements from the structured light camera are accurately mapped to the world coordinate system, enabling precise and consistent calibration and reconstruction.

### 2.2. Hand–Eye Calibration Algorithm

**Figure 2:**
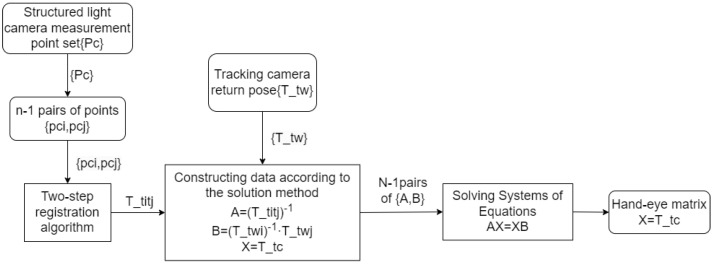
Calibration algorithm flow.

As shown in Figure 2, the raw data for the calibration algorithm proposed in this paper includes measurement data of non-specific 3D targets and the corresponding tracking data. The computation of the hand–eye matrix $T_{c}^{t}$ is completed in three steps as follows: registration, equation construction, and solution.

### 2.3. Construction of Calibration Equation

**Figure 3:**
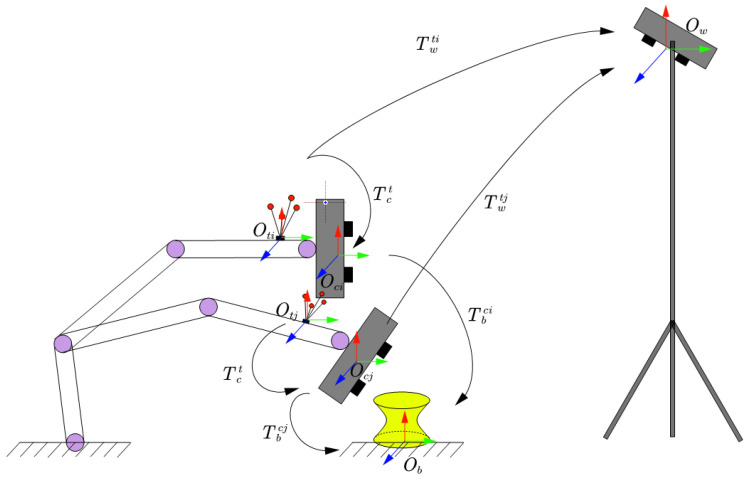
Multi-pose calibration measurement.

As shown in Figure 3, the robot is used to drive the structured light camera to capture the point set $P_{c}=\left\{p_{c1},p_{c2},\ldots ,p_{cj}\right\}$ of the arbitrary 3D target in the structured light camera coordinate system $\left\{O_{c}\right\}$. Simultaneously, the tracking camera obtains the pose $\left\{T_{t}^{w}\right\}$ of the tracking target *t* in the world coordinate system $\left\{O_{w}\right\}$ for each acquisition.

At positions *i* and *j*, the homogeneous transformation matrices between the world coordinate system $O_{w}$ and the structured light camera coordinate system $O_{c}$ are denoted as $T_{ci}^{w}$ and $T_{cj}^{w}$, respectively. These matrices satisfy the following relationships:

(2) $$\left\{\begin{array}{c} T_{ci}^{W}={T_{w}^{ti}}^{-1}\cdot T_{c}^{t}\\ T_{cj}^{W}={T_{w}^{tj}}^{-1}\cdot T_{c}^{t} \end{array}\right.$$

The homogeneous transformation matrix between the structured light camera coordinate systems at the two positions, denoted as $T_{cj}^{ci}$, satisfies the following:

(3) $$T_{cj}^{ci}={T_{cj}^{w}}^{-1}\cdot T_{ci}^{w}$$

By combining Equations (2) and (3), we obtain the following:

(4) $$T_{cj}^{ci}={\left({T_{w}^{tj}}^{-1}\cdot T_{c}^{t}\right)}^{-1}\cdot \left({T_{w}^{ti}}^{-1}\cdot T_{c}^{t}\right)={T_{c}^{t}}^{-1}\cdot T_{w}^{tj}\cdot {T_{w}^{tj}}^{-1}\cdot T_{c}^{t}$$

Here, $T_{tj}^{ti}$ represents the registration result of the point cloud of the stereo calibration target at the two positions, and it satisfies the following:

(5) $$T_{tj}^{ti}=T_{w}^{ti}\cdot {T_{w}^{tj}}^{-1}={\left(T_{w}^{tj}\cdot {T_{w}^{ij}}^{-1}\right)}^{-1}$$

Substituting Equation (5) into Equation (4), we obtain the following:

(6) $$T_{cj}^{ci}={T_{c}^{t}}^{-1}\cdot {T_{tj}^{ti}}^{-1}\cdot T_{c}^{t}$$

(7) $${T_{tj}^{ti}}^{-1}\cdot T_{c}^{t}=T_{c}^{t}\cdot T_{cj}^{ci}$$

In Equation (7), let ${T_{tj}^{ti}}^{-1}=A$, $T_{cj}^{ci}=B$, and $T_{c}^{t}=X$. For a pair of 3D calibration target measurements and their corresponding tracked target poses, the structure of the robot hand–eye matrix $T_{c}^{t}$ can be formulated as the equation AX=XB.

**Figure 4:**
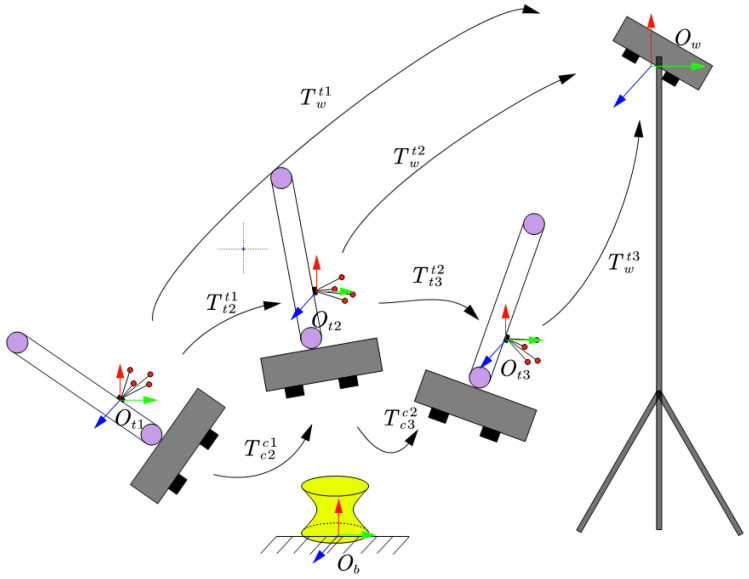
Multi-pose measurement transformation matrix relationship.

As shown in Figure 4, this process is extended to perform 3D scanning of the stereo calibration target at multiple poses, obtaining multiple sets of point cloud data in the structured light camera coordinate system, as well as the corresponding tracked target poses $T_{w}^{ti}$. Sequentially, the absolute poses of the tracked target in the world coordinate system, $T_{w}^{ti}$ and $T_{w}^{tj}$, are used to obtain n−1 pairwise relative pose homogeneous matrices $T_{tj}^{ti}$. Sequentially, adjacent point clouds are pairwise calibrated, resulting in n−1 homogeneous matrices $T_{cj}^{ci}$ from *n* sets of data. After obtaining multiple AX=XB matrix equations, quaternion decomposition and the least squares method are used to solve the system of matrix equations.

In addition to the direct derivation of the transformation matrix equation system mentioned earlier, this calibration process can also utilize existing data and the spatial relationships contained within to convert parameters into a homogeneous transformation matrix of the 3D calibration target in the structured light camera coordinate system, similar to the calibration method described previously. For any point $\left(P_{ci},P_{ci},P_{ci}\right)$ on the 3D calibration target measured by the structured light camera at positions *i* and *j*, the transformation relationship $T_{ti}$ between the point clouds after registration satisfies the following:

(8) $$P_{ci}=T_{ti}\cdot P_{cj}$$

In the world coordinate system, any point *p* on the 3D calibration target satisfies the following in the structured light camera coordinate system:

(9) $$P_{ci}=T_{wi}\cdot P_{w}$$

Combining these equations, we obtain the following:

(10) $$T_{wj}\cdot P_{w}=T_{ti}\cdot T_{wi}\cdot P_{w}$$

Thus, the following holds:

(11) $$T_{wj}=T_{ti}\cdot T_{wi}$$

Here, $\left(T_{wi},T_{wj}\right)$ represents the poses of the camera in the world coordinate system. The transformation of the structured light camera’s pose in space is the inverse of the point cloud transformation, expressed as follows:

(12) $$T_{wj}=T_{ti}^{-1}$$

After indirectly obtaining the position relationship of the camera in the world coordinate system through point cloud registration, variables can be constructed to satisfy the data input requirements of traditional hand–eye calibration, and the corresponding algorithm can be invoked. The inputs are the homogeneous matrix $T_{\mathrm{gripper}2\mathrm{base}}$ from the robot flange to the world coordinate system and the homogeneous matrix $T_{\mathrm{Target}2\mathrm{cam}}$ from the target to the camera. The output is the hand–eye matrix.

In the system constructed in this work, the homogeneous matrix $T_{vw}$ of the tracked target in the world coordinate system of the tracking camera is equivalent to the homogeneous matrix $T_{\mathrm{gripper}2\mathrm{base}}$ from the robot flange to the world coordinate system. The virtual camera coordinate system is defined as the coordinate system of the first stereo calibration target, and its matrix $T_{\mathrm{gripper}2\mathrm{base}}$ is the identity matrix *I*. For the *k*-th stereo calibration target, the homogeneous matrix $T_{\mathrm{gripper}2\mathrm{base}}$ relative to the virtual camera coordinate system is the registration result $T_{ck-1}$ of the stereo calibration target point cloud, which can also be expressed as the product of the sequential registration results as follows:

(13) $$T_{\mathrm{gripper}2\mathrm{base}}={T_{ci}^{ck}}^{-1}={\prod_{i=k}^{n}T_{ci}^{c1}}^{-1}$$

By constructing the virtual camera coordinate system using the relative relationships of the stereo calibration targets obtained through registration, the transformation of homogeneous matrices can be achieved, making it compatible with existing hand–eye matrix calculation functions. This enables the implementation of the robot hand–eye calibration calculation based on arbitrarily shaped 3D calibration targets as designed in this work.

### 2.4. Multi-Step Registration Algorithm

The hand–eye calibration method proposed in this paper that is based on arbitrary 3D targets relies on the scanning and calibration of the target’s point cloud. It requires precise calibration calculations for point clouds with significant initial pose differences and partially non-overlapping regions to accurately determine the spatial relationship of the target’s measured area. This, in turn, enables the determination of the spatial relationship of the camera.

**Figure 5:**
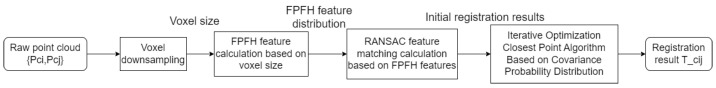
Multi-step registration algorithm.

As shown in Figure 5, to meet the requirements of the calibration algorithm, this paper designs a registration method that includes the following two steps: coarse registration based on local geometric feature calculation and matching, and fine registration based on an improved ICP algorithm, achieving the preliminary processing of the collected data.

The RANSAC (Random Sample Consensus) algorithm is used to calculate the local feature distribution of point clouds based on a FPFH (Fast Point Feature Histogram) [22], enabling the registration of the source point cloud with the target point cloud. First, the voxel size for voxel filtering is set as the dimension for voxel downsampling, along with the radius for normal vector estimation and the search radius for FPFH feature extraction. For the downsampled voxel grid point cloud, the voxel size is used as the basic unit for the KD-tree search radius, and the nearest neighbors within each point’s neighborhood are searched to estimate the normal vectors.

The estimated normal vectors of each point are used to compute the SPFH (Simplified Point Feature Histogram) for that point. The voxel size from the voxel downsampling is set as the basic unit for the neighborhood point search radius. For each point, it forms a point pair $\left(p_{t},p_{s}\right)$ and their estimated normal vectors $\left(n_{t},n_{s}\right)$, a coordinate system $O_{p}\left(u,v,w\right)$ is constructed, satisfying:For a point pair $\left(p_{t},p_{s}\right)$ and their estimated normal vectors $\left(n_{t},n_{s}\right)$, and a coordinate system $O_{p}\left(u,v,w\right)$ is constructed, satisfying the following:

(14) $$\left\{\begin{array}{c} u=n_{s}\\ v=\frac{\left(p_{t}-p_{s}\right)}{\left||(p_{t}-p_{s})|\right|}\times u\\ w=u\times v \end{array}\right.$$

In the coordinate system, the pairwise features α, ϕ, and θ for the point pair are calculated as follows:

(15) $$\left\{\begin{array}{c} \alpha =vs.\cdot n_{t}\\ \phi =u\cdot \frac{\left(p_{t}-p_{s}\right)}{d}\\ \arctan(w\cdot n_{t},u\cdot n_{t}) \end{array}\right.$$

where *d* represents the Euclidean distance between the point pair as follows:

(16) $$d=\left|\right|p_{t}-p_{s}{\left|\right|}_{2}$$

Through the above calculations, for each point $p_{s}$ and its neighboring points $\left\{p_{t}\right\}$, the *n* point pairs form the SPFH for each point, which can also be expressed as follows:

(17) $$SPFH\left(p_{t}\right)=\left[h_{\alpha }\left(p_{t}\right),h_{\phi }\left(p_{t}\right),h_{\theta }\left(p_{t}\right)\right]$$

where $h_{\alpha }\left(p_{t}\right)$, $h_{\phi }\left(p_{t}\right)$, and $h_{\theta }\left(p_{t}\right)$ are histograms based on the values of α, ϕ, and θ.

For each point, the SPFH is weighted and summed, incorporating the information from neighboring points into the final feature parameters of the point. For point $p_{t}$, its $\mathrm{FPFH}(p_{t})$ consists of the following two parts: the first part is its own $\mathrm{SPFH}(p_{t})$, and the second part is the weighted average of the *k* neighboring points using the inverse distance as the weight. Thus, the $\mathrm{FPFH}(p_{t})$ can be expressed as follows:

(18) $$FPFH\left(p_{t}\right)=SPFH\left(p_{t}\right)+\frac{1}{k}\sum_{i=1}^{k}\frac{1}{d(p_{t},p_{i})}$$

Through the above calculations, the FPFH of the source point cloud and the target point cloud are obtained. Randomly select *N* feature point pairs $P_{s},P_{t}$ from the source and target point clouds where the FPFH matches. First, we use SVD (Singular Value Decomposition) to estimate the rotation part of the rigid transformation matrix for these point pairs. Centroid normalization is performed on these points as follows:

(19) $$\left\{\begin{array}{c} P_{t}^{\prime }=P_{t}-\frac{1}{N}\sum_{i=1}^{N}P_{t,i}\\ P_{s}^{\prime }=P_{s}-\frac{1}{N}\sum_{i=1}^{N}P_{s,i} \end{array}\right.$$

For the centroid-normalized point sets $P_{s}^{\prime }$ and $P_{t}^{\prime }$, the covariance matrix *H* is calculated as follows:

(20) $$H=\sum_{i=1}^{N}P_{s}^{\prime }\left[i\right]⨂P_{t}^{t}\left[i\right]$$

where ⨂ denotes the outer product.

The covariance matrix *H* is decomposed using SVD as follows:

(21) $$H=USV^{T}$$

From this, the rotation transformation *R* for the point sets $P_{s}^{\prime }$ and $P_{t}^{\prime }$ is obtained as follows:

(22) $$R=V\cdot U^{T}$$

Based on the rotation transformation *R*, the origin displacement of the point sets $P_{s}^{\prime }$ and $P_{t}^{\prime }$ is calculated to obtain the translation transformation *t* as follows:

(23) $$t=\frac{1}{N}\sum_{i=1}^{N}P_{t,i}-R\cdot \frac{1}{N}\sum_{i=1}^{N}P_{s,i}$$

Combining *R* and *t* yields the homogeneous transformation matrix *T*, as follows:

(24) $$T=\left[\begin{array}{cc} R & t\\ 0 & 1 \end{array}\right]$$

Based on the initially calculated homogeneous transformation matrix *T*, the distance difference between the feature-matched corresponding points is computed. If the difference is below the threshold, the global feature registration is completed. If the difference exceeds the threshold, the above steps are repeated, and the final homogeneous transformation matrix *T* is obtained by cumulative multiplication and combination.

For the point cloud after coarse registration based on RANSAC and FPFH features, the optimized Iterative Closest Point (ICP) algorithm [23] is executed to further improve registration accuracy, achieving results that meet the precision requirements of the calibration process.

Using the registration result from RANSAC as the initial transformation, for each point $p_{i}$ in the source point cloud and $p_{j}$ in the target point cloud, the Mahalanobis distance $d(p_{i},p_{j})$ is calculated as the error metric as follows:

(25) $$d\left(p_{i},p_{j}\right)={\left(p_{i}-p_{j}\right)}^{T}\cdot {\left(C_{i}+C_{j}\right)}^{-1}\cdot \left(p_{i}-p_{j}\right)$$

where $C_{i}$ and $C_{j}$ are the covariance matrices of the source and target points within their neighborhoods. For any point $p_{i}$ in the point cloud, its covariance is the outer product of the coordinate differences between $p_{i}$ and its neighboring points $q_{j}$, expressed as follows:

(26) $$C_{i}=\frac{1}{N}\sum_{j=1}^{n}{\left(q_{j}-p_{i}\right)}^{T}\cdot \left(q_{j}-p_{i}\right)$$

where *n* is the neighborhood size.

For the registration step, the target transformation matrix *T* is as follows:

(27) $$T=\left[\begin{array}{cc} R & t\\ 0 & 1 \end{array}\right]\in SE\left(3\right)$$

The Mahalanobis distance-based error E(T) between the target point set {p} and the source point set {q} is expressed as follows:

(28) $$E\left(T\right)=\sum_{i=1}^{n}{\left(q_{i}-Tp_{i}\right)}^{T}\cdot C_{i}^{-1}\cdot \left(q_{i}-Tp_{i}\right)$$

Expanding this gives the following:

(29) $$E\left(R,t\right)=\sum_{i=1}^{n}{\left(q_{i}-\left(Rp_{i}+t\right)\right)}^{T}\cdot C_{i}^{-1}\cdot \left(q_{i}-\left(Rp_{i}+t\right)\right)$$

The Gauss–Newton method is used to perform a first-order Taylor expansion of the Mahalanobis distance error E(T) for iterative optimization. First, the transformation matrix *T* is parameterized as a vector *x* as follows:

(30) $$x={\left[\omega^{T},t^{T}\right]}^{T}$$

where $\omega \in R^{3}$ is the Lie algebra representation of R∈SO(3).

For each point pair $\left(p_{i},q_{i}\right)$, the corresponding residual $e_{i}\left(x\right)$ is as follows:

(31) $$e_{i}\left(x\right)=q_{i}-\left(Rp_{i}+t\right)$$

The weighted sum of squared residuals E(x) is expressed as follows:

(32) $$E\left(x\right)=\sum_{i=1}^{n}e_{i}^{T}C_{i}^{-1}e_{i}$$

The residual $e_{i}\left(x\right)$ is Taylor-expanded to approximate the nonlinear error as follows:

(33) $$e_{i}\left(x+\Delta x\right)\approx e_{i}\left(x\right)+J_{i}\Delta x$$

where $\Delta x={\left[\Delta \omega^{T},\Delta t^{T}\right]}^{T}$ is the parameter increment, and $J_{i}=\frac{\partial e_{i}}{\partial x}\in R^{3\times 6}$ is the Jacobian matrix of the error $e_{i}$ with respect to parameters *x*. The rotation derivative $\frac{\partial e_{i}}{\partial \omega }$ and translation derivative $\frac{\partial e_{i}}{\partial t}$ are expressed as follows:

(34) $$\frac{\partial e_{i}}{\partial \omega }=-I_{3\times 3}$$

(35) $$\frac{\partial e_{i}}{\partial t}=\frac{\partial Rp_{i}}{\partial t}\approx -{\left(Rp_{i}\right)}^{\wedge }$$

where ${\left(\cdot \right)}^{\wedge }$ denotes the cross-product matrix of the vector. Through Taylor expansion, the objective function E(x) can be approximated as a least squares problem as follows:

(36) $$E\left(x+\Delta x\right)\approx \sum_{i=1}^{n}{\left(e_{i}+J_{i}\Delta x\right)}^{T}C_{i}^{-1}\left(e_{i}+J_{i}\Delta x\right)$$

(37) $$E=\sum_{i=1}^{n}\left(e_{i}^{T}C_{i}^{-1}e_{i}+2e_{i}^{T}C_{i}^{-1}J_{i}\Delta x+\Delta x^{T}J_{i}^{T}C_{i}^{-1}J_{i}\Delta x\right)$$

Ignoring higher-order terms, it is approximated as follows:

(38) $$E\approx \sum_{i=1}^{n}e_{i}^{T}C_{i}^{-1}e_{i}+2\Delta x\sum_{i=1}^{n}J_{i}^{T}C_{i}^{-1}e_{i}+\Delta x^{T}\left(\sum_{i=1}^{n}J_{i}^{T}C_{i}^{-1}J_{i}\right)\Delta x$$

To minimize *E*, the derivative with respect to Δx is taken and set to zero, yielding the Gauss–Newton equation, expressed as follows:

(39) $$\left(\sum_{i=1}^{n}J_{i}^{T}C_{i}^{-1}J_{i}\right)\Delta x=-\sum_{i=1}^{n}J_{i}^{T}C_{i}^{-1}e_{i}$$

where $H=\sum_{i=1}^{n}J_{i}^{T}C_{i}^{-1}J_{i}$ is the Gauss–Newton Hessian matrix, and $g=-\sum_{i=1}^{n}J_{i}^{T}C_{i}^{-1}e_{i}$ is the gradient vector.

After obtaining the linear system of equations for Δx, an approximate solution is computed iteratively until $|\Delta x_{k}|$ is below the threshold ϵ. The cumulative Δx corresponds to ΔT, yielding the final transformation matrix *T*.

## 3. Experiment

### 3.1. Experimental Platform and Evaluation Criteria

#### 3.1.1. Experimental Platform

**Figure 6:**
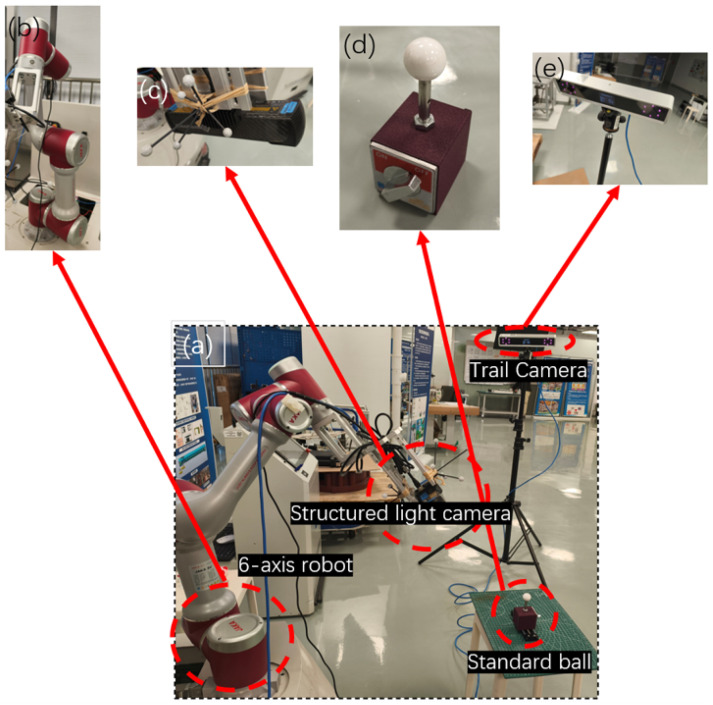
Experimental hardware system. (**a**) Experimental system, (**b**) six-axis robotic arm, (**c**) structured light camera, (**d**) ceramic standard ball, and (**e**) tracking camera.

We build the experimental hardware system as shown in Figure 6. The experiment uses the JAKA Zu7 collaborative robot to drive the structured light camera and the tracking target, with a repeatability of ±0.02 mm. The PhoXi 3D Scanner S structured light camera is used for 3D data scanning, with a scanning range of 384–520 mm, point spacing of 0.174 mm, and calibration accuracy of 0.050 mm. The AITS D-Series Smart tracking camera is used to track the target on the robotic arm to obtain the end-effector pose, with a tracking distance of 0.5–2.0 m and tracking accuracy of 0.15–0.30 mm. Arbitrarily selected objects are used as the calibration targets for hand–eye calibration experiments. After completing system calibration, a ceramic standard sphere with a diameter of 25±0.001 mm is used for calibration experiments. The experiments include 3D reconstruction error experiments and distance measurement error experiments.

#### 3.1.2. Evaluation Criteria

The 3D reconstruction error refers to controlling the robot to measure the point cloud of a standard sphere placed at a fixed position from different locations. The sphere center position $O_{c}$ in the structured light camera coordinate system is obtained through fitting. Using the hand–eye matrix from the previous calibration experiment and the transformation matrix of the structured light camera in the world coordinate system obtained from the tracking camera, the sphere center positions scanned from different locations are unified into the world coordinate system of the tracking camera. The position error of the fixed standard sphere center unified into the world coordinate system under different structured light camera positions is the metric for evaluating the 3D reconstruction error of the robot measurement system.

The distance measurement error refers to controlling the robot system to remain stationary and using a slide rail with a movement accuracy of 0.1 mm to measure the point cloud of the standard sphere before and after movement. After sphere center identification and coordinate system transformation, the error between the distance of the sphere center positions in the world coordinate system and the actual measured distance is the distance measurement error.

### 3.2. Experimental Procedures

**Figure 7:**
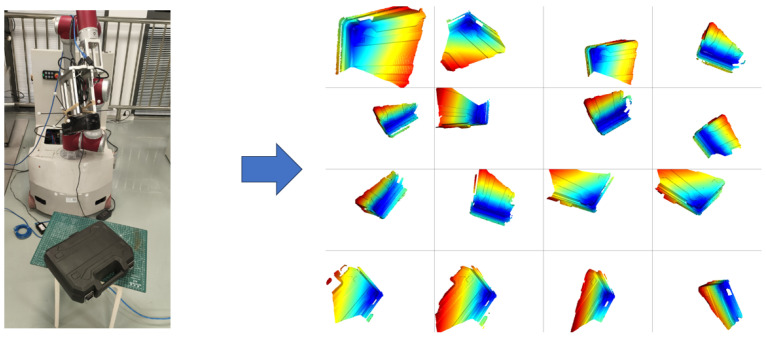
Calibration data collection.

According to the calibration process described in the methodology, the proposed calibration method requires experimental data consisting of a set of 3D point cloud scans of an object and a corresponding set of tracking poses. As shown in Figure 7, the robot-controlled scanning camera is used to scan a toolbox as the 3D target at different positions, resulting in 16 sets of corresponding point clouds and tracking poses.

**Figure 8:**
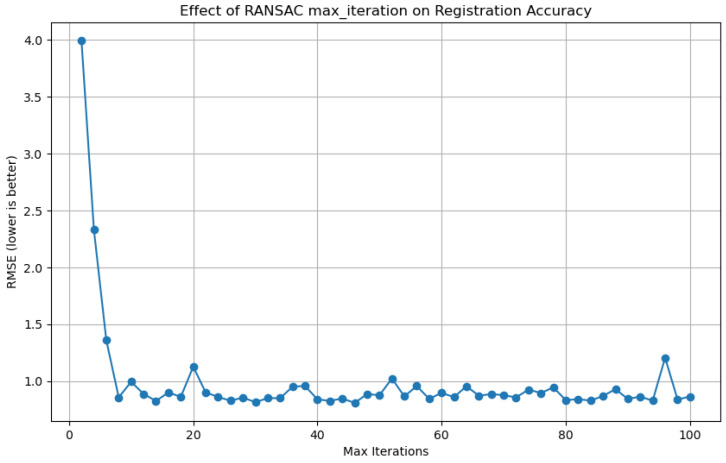
Influence of RANSAC iteration count on the RMSE of global registration.

According to the registration method proposed in this study, the number of iterations in the initial stage of global registration significantly influences the registration accuracy. As illustrated in Figure 8, during the registration of the first two sets of target point clouds, the impact of the number of RANSAC iterations on the RMSE is demonstrated. It can be observed from Figure 8 that a relatively small number of iterations is sufficient for achieving stable registration performance, with the RMSE reaching its optimal and stable value after approximately 8 iterations.

**Figure 9:**
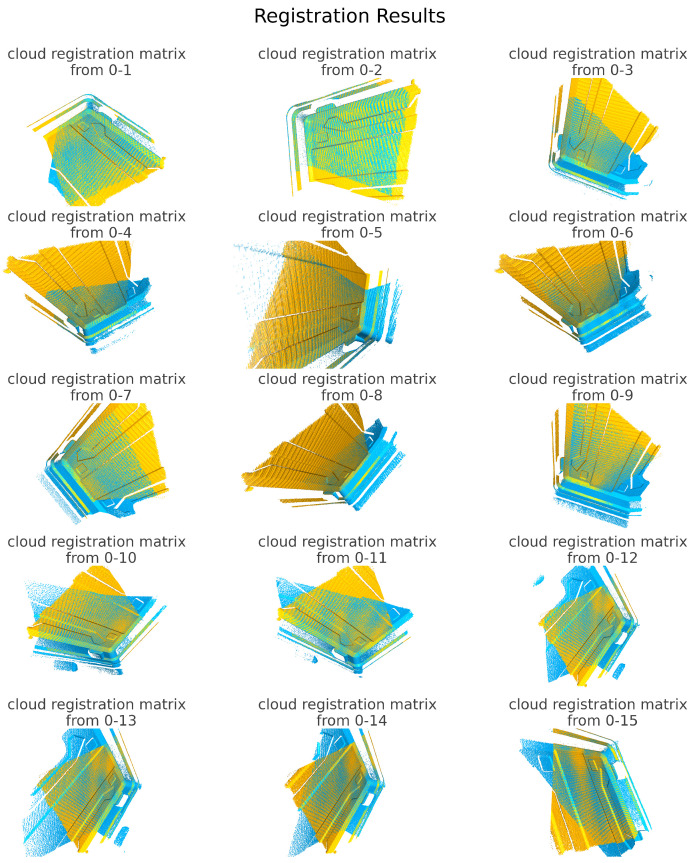
Target pose registration.

As shown in Figure 9, following the registration method described in Section 2.2, 15 sets of registrations are performed on the 16 sets of point clouds, resulting in 15 transformation matrices $\left\{T_{t1}^{ti}\right\}$ that represent the relative poses of point clouds 2–16 with respect to point cloud 1.

**Figure 10:**
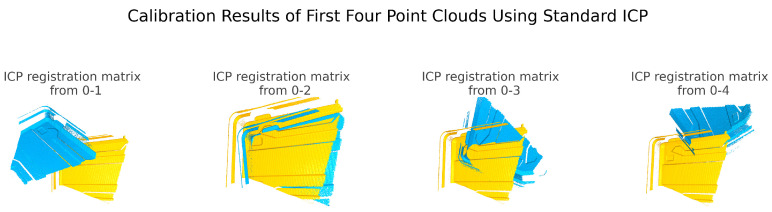
Pointcloud registration by standard ICP (First Four Sets).

Because the calibration method proposed in this paper involves the registration of the same object under significant pose variations, the standard ICP registration algorithm is prone to becoming stuck in local optima and failing to achieve correct registration due to large initial pose differences and small overlapping areas. However, since this paper introduces a global registration algorithm and a covariance-optimized ICP algorithm, it can effectively achieve point cloud registration in such scenarios. Figure 10 shows the registration of the first four pairs of point clouds in this experiment using the standard ICP method, and most of the point clouds failed to achieve correct registration.

**Figure 11:**
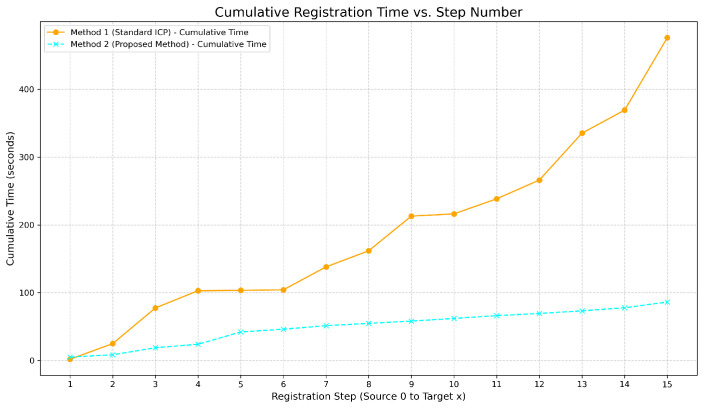
Comparison of registration time.

To compare the computation time of the standard ICP method and the proposed registration method under this dataset, the experiment was conducted on a laptop equipped with an AMD Ryzen 7 4800U processor. This processor is based on the Zen 2 architecture, featuring eight physical cores and 16 threads, a base clock frequency of 1.8 GHz, a maximum boost clock frequency of up to 4.2 GHz, and 8 MB of L3 cache. As shown in Figure 11, due to the introduction of global registration and covariance optimization, the proposed method can consistently maintain the computation time for each step at approximately 4 s. In contrast, standard ICP is prone to becoming trapped in local optima, which increases computation time. The proposed registration method reduces computation time by 81.87.

**Figure 12:**
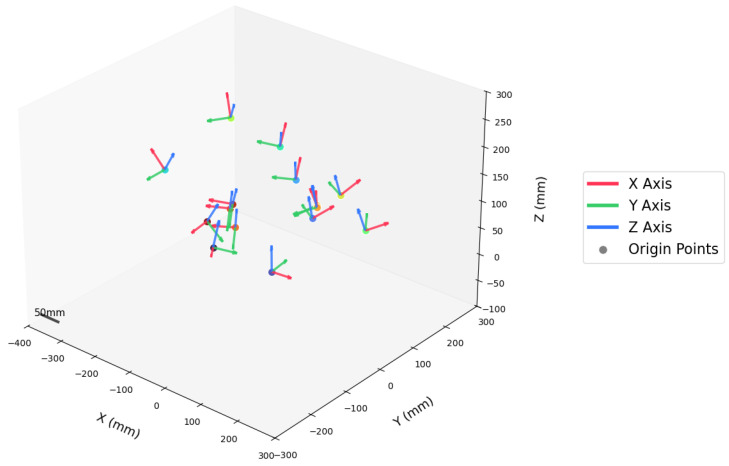
Spatial distribution of pose data.

The spatial poses contained in this set of data are illustrated in Figure 12. Simultaneously, during each scan, the tracking camera obtains the transformation matrices $\left\{T_{w}^{ti}\right\}$ of the tracking target on the structured light camera in the world coordinate system.

The measured values are input into the hand–eye calibration algorithm proposed in Section 2, yielding the transformation matrix of the tracking target relative to the structured light camera coordinate system, i.e., the hand–eye matrix $T_{c}^{t}$, with the following value:

$$T_{c}^{t}=\left[\begin{array}{cccc} 0.66942031 & -0.69956424 & 0.24997263 & 60.8915365\\ -0.64794354 & -0.71441506 & -0.26415958 & -122.40444553\\ 0.36338081 & 0.01486563 & -0.93152209 & -15.22424098\\ 0 & 0 & 0 & 1 \end{array}\right]$$

### 3.3. Three-Dimensional Reconstruction Error

**Figure 13:**
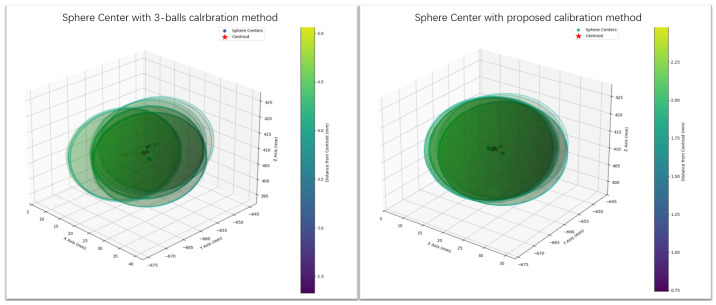
Three-Dimensional reconstruction error.

**Figure 14:**
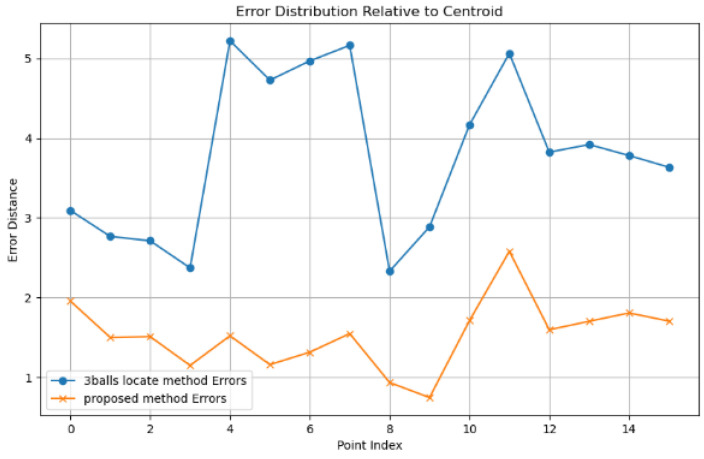
Three-Dimensional reconstruction error distribution.

This paper uses the three-standard-sphere calibration method as the control group. The robot hand–eye matrix is obtained by following the calibration process proposed in Section 2.2, using 16 sets of coordinate systems composed of three standard spheres. Figure 13 shows the visualization results of the 3D reconstruction error validation for the ceramic standard sphere using the calibration results obtained in the previous chapter. The mean error of the 16 sets of sphere center coordinates is 1.53 mm, with a standard deviation of 0.42. In the control experiment under the same hardware system, as shown in Figure 14, the 3D reconstruction results of the ceramic standard sphere using the proposed method are compared with those obtained using the three-sphere-center coordinate system method. The mean error of the 16 sets of sphere center coordinates for the latter is 3.79 mm, with a standard deviation of 0.99. The experimental results demonstrate that the proposed method, compared to the three-point coordinate system method, achieves a 59.62% reduction in mean error and a 57.54% reduction in standard deviation, while requiring lower precision for the calibration object and simpler execution steps.

A comparison of the 3D reconstruction position errors between the method proposed in this paper, the three-standard-sphere coordinate system method, and other research methods is shown in Table 1.

The 3D reconstruction data demonstrates that the proposed method in this paper achieves the smallest mean error in the calibration of structured light cameras for arbitrary 3D objects. Compared to the experimental results obtained using laser scanner devices, the error is significantly larger. While the evaluation metric used in this paper is a 3D reconstruction error, recent learning-based methods such as EasyHeC [27] and EasyHeC++ [28] report their performance in terms of translation error during hand–eye calibration. According to the original publications, EasyHeC(2023) achieves a mean translation error of 2.06 mm, and EasyHeC++(2024) improves this to 1.35 mm, both under specific experimental setups involving fiducial targets and high-performance industrial cameras. In comparison, the 3D reconstruction error reported in this paper is 1.53 mm, which inherently incorporates the calibration error as one of its components. This indicates that the proposed method achieves an overall performance comparable to or better than EasyHeC and EasyHeC++ in practical applications, despite using low-cost arbitrary 3D targets and a more accessible experimental setup. The results highlight the flexibility and applicability of the proposed method in scenarios where specialized hardware and fiducial targets are unavailable.

### 3.4. Distance Measurement Error

**Figure 15:**
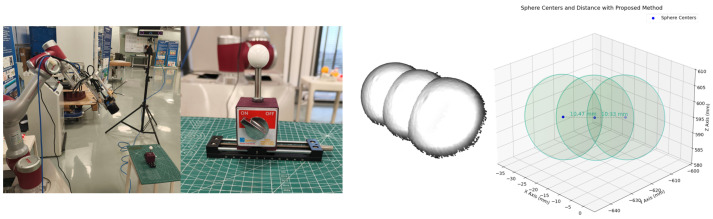
Distance measurement error.

As shown in Figure 15, for a set of linear sliders, the point clouds of the spherical surface obtained at positions of −10.0 mm, 0.0 mm, and 10.0 mm have sphere center distances of 10.47 mm and 10.31 mm, respectively, with an average distance measurement error of 0.39 mm. The sources of error mainly include the calibration error of the structured light camera (0.05 mm), the tracking error of the tracking camera (0.15 mm), potential positional errors in the sphere center fitting calculation, and cumulative errors that may arise during the computational process.

## 4. Conclusions

In robotic measurement systems, hand–eye calibration is an essential task. For the calibration of 3D cameras, it is often necessary to perform complex intrinsic camera calibration, followed by calibration using a calibration board with specific patterns or relying on calibration objects with high machining precision, such as standard spheres. To simplify the calibration process, this paper proposes a hand–eye calibration method based on a highly adaptable and high-precision registration approach, using arbitrary 3D targets. The proposed method employs arbitrary spatial objects as 3D targets, obtains the relative positions of each captured data through multi-step registration, and indirectly derives the spatial relationships between cameras, thereby establishing a system of linear equations for the hand–eye relationship. By solving these equations, the hand–eye relationship of the camera system is obtained. Experimental results show that, compared to conventional calibration methods based on standard spheres for establishing spatial coordinate systems, the proposed method achieves higher precision and greater spatial coverage in 3D reconstruction error experiments, as well as higher accuracy in distance measurement error experiments.

However, the proposed method still faces some robustness and precision issues, primarily due to the reliance of the registration step on the high precision and resolution of the 3D camera itself, the dependence on tracking accuracy during data collection, and the residuals generated by the hand–eye relationship solving algorithm when the spatial relationships covered by the raw data are limited. Developing registration algorithms with higher overall feature recognition capabilities or improving the performance of measurement hardware in the system could help enhance the accuracy of the calibration results and the overall precision of the measurement system. In this paper, the experimental data acquisition was performed using a manual robot control method, which is relatively labor-intensive and time-consuming. To improve the automation of this process, further research can be conducted on the arrangement of acquisition points and the automatic planning of calibration data collection. The 3D target required by the proposed method needs to satisfy non-centrosymmetric geometric features. Furthermore, due to the precision limitations imposed by the 3D camera’s resolution, using excessively small 3D targets may reduce the calibration accuracy. Developing registration algorithms with stronger spatial feature extraction capabilities could further enhance the applicability of this method.

## Figures and Tables

**Table 1:** Mean and distribution of 3D reconstruction errors for different methods.

Method	Camera Type	Target Type	Mean Error (mm)	Standard Deviation
Three-Ball System Method	Structured Light	Three Standard Spheres	(*X*: 2.77, *Y*: 1.49, *Z*: 1.47) 3.79	0.99
Zhe’s Method [24]	Line Laser	2D Calibration Board	(*X*: 1.334, *Y*: 0.511, *Z*: 0.925) 1.855	-
Murali’s Method [25]	Laser Profiler	Arbitrary 3D Object	(*X*: 0.701, *Y*: 0.443, *Z*: 0.366) 0.906	-
Peter’s Method [26]	Structured Light	Arbitrary 3D Object	1.77	-
Proposed Method	Structured Light	Arbitrary 3D Object	(*X*: 1.10, *Y*: 0.60, *Z*: 0.66) 1.53	0.42

## Data Availability

The data presented in this study are available on request from the corresponding author.
